# A novel case of prosthetic joint infection due to Clostridioides difficile successfully suppressed with oral doxycycline

**DOI:** 10.1099/acmi.0.000943.v3

**Published:** 2025-09-19

**Authors:** Holly Jordan, Rikki Graham, Sanmarie Schlebusch, Aileen Oon, Hemalatha Varadhan, Syeda Naqvi

**Affiliations:** 1 Department of Infectious Diseases, John Hunter Hospital, Newcastle, NSW, Australia; 2 School of Medicine and Public Health, University of Newcastle, Newcastle, NSW, Australia; 3 Public Health Microbiology & Queensland Public Health and Infectious Diseases Reference Genomics, Public and Environmental Health Reference Laboratories, Pathology Queensland, Brisbane, QLD, Australia; 4 University of Queensland (UQ) Medical School, Brisbane, QLD, Australia; 5 New South Wales Health Pathology, Hunter, NSW, Australia

**Keywords:** antibiotic stewardship, *Clostridium difficile*, prosthetic joint infection, whole-genome sequencing

## Abstract

Extra-colonic infections caused by *Clostridioides difficile* are exceptionally rare, with prosthetic joint infections (PJIs) comprising only a small fraction of the reported cases. Moreover, there is limited guidance on the optimal management of such infections. We present the case of a 76-year-old man who developed a left hip PJI due to *C. difficile* 6 weeks after undergoing surgical revision for a periprosthetic fracture. Given the complexity of the case, curative surgical intervention was not considered feasible. The patient was treated with repeated debridement, intravenous vancomycin and oral (PO) metronidazole, followed by successful suppression with PO doxycycline – a novel therapeutic approach not previously documented. To date, only seven cases of *C. difficile*-associated PJI have been reported in the literature; this is the first known instance in which suppression of a *C. difficile* PJI has been achieved and the first to utilize whole-genome sequencing for further analysis of the isolate.

## Data Summary

All data associated with this work are reported within the article.

## Case presentation

**Fig. 1.**
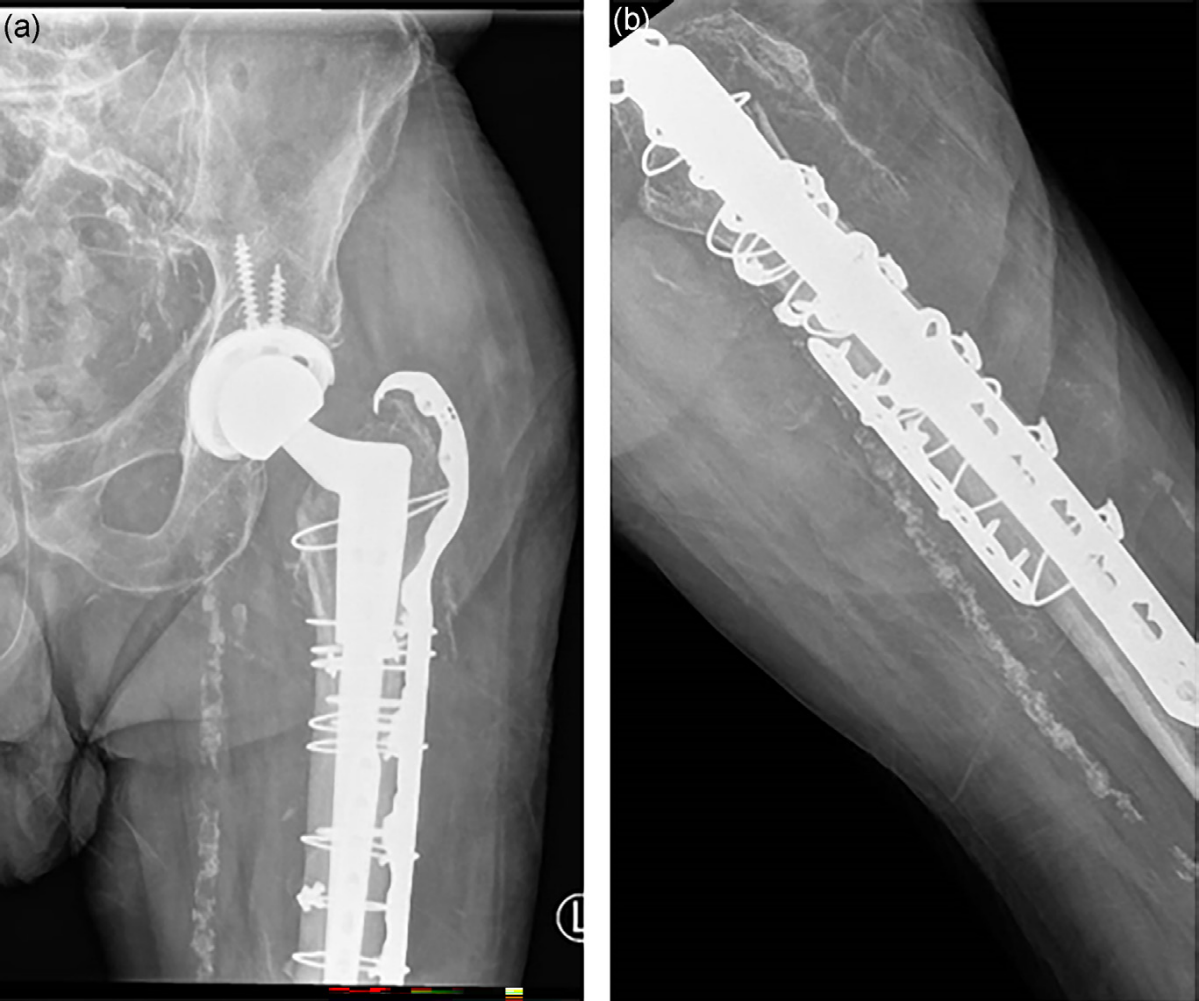
Post-operative L) hip x-ray following (a) revision and (b) fixation.

A 76-year-old man, with a past medical history of ischaemic heart disease, peripheral vascular disease and excessive alcohol consumption, presented to a regional hospital after falling at home and sustaining a left neck of femur fracture. He underwent a non-elective left total hip replacement (THR) and was discharged back home after a short inpatient stay. During his index admission, he received 7 days of oral (PO) amoxicillin for a urinary tract infection caused by *Proteus mirabilis*. Five months post-discharge, the patient experienced a subsequent fall and sustained a closed periprosthetic left femur fracture. He was transferred to the affiliated tertiary referral hospital and underwent a revision of his left THR and femoral fixation (Fig. 1); he received surgical antimicrobial prophylaxis with intravenous (IV) cefazolin as per the institution’s local guidelines. At 48 h post-operatively, the patient developed profuse, watery diarrhoea, and *Clostridioides difficile* was subsequently detected in the patient’s stool using the Cepheid, Sunnyvale, California Xpert^®^ *C. difficile/Epi* assay – a rapid, automated, real-time PCR *in vitro* diagnostic test that detects the toxin B gene (*tcdB*), the binary toxin gene (CDT) and the single-base-pair deletion at nucleotide 117 in the *tcdC* gene [1]. He was treated with 10 days of PO fidaxomicin, followed by a PO vancomycin taper due to severe and protracted diarrhoea. A combination of premorbid comorbidities, frailty and acute illness post-operatively rendered the patient immobile for a prolonged period following his revision. During this time, the patient had several episodes of faecal incontinence and soiling in close proximity to the surgical site that prompted more frequent dressing changes than expected.

**Fig. 2.**
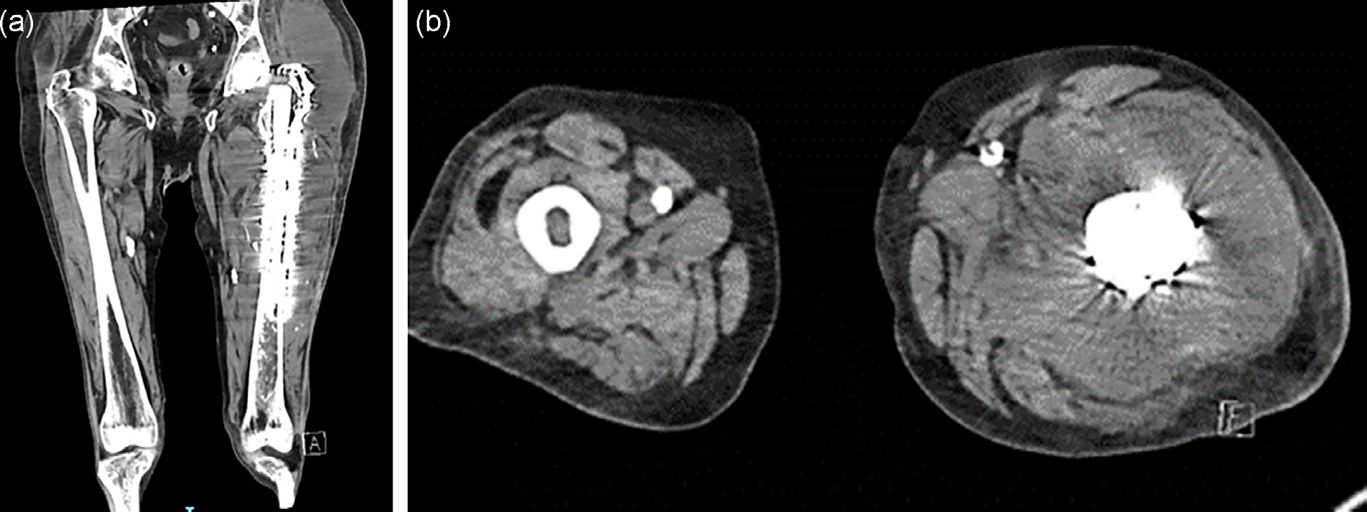
(a) Coronial and (b) axial CT images showing large fluid collection surrounding femoral stem and fixation.

Five weeks later, the patient reported new and progressive left hip pain. Laboratory evaluation revealed a rising C-reactive protein (CRP), peaking at 294 mg l^−1^, and a white blood cell count of 30.1×10^9^ cells per litre with a predominant left-shifted neutrophilia of 17.6×10^9^ cells per litre. The patient was commenced empirically on IV cefazolin 2 g three times a day (TDS), and a computed tomography (CT) scan of the left femur was performed, which demonstrated a large fluid collection with associated subcutaneous stranding surrounding the left femoral stem and fixation (Fig. 2). The patient underwent an ultrasound-guided percutaneous aspiration of the collection, followed by operative debridement and washout, without revision or removal of hardware. Intraoperatively, massive amounts of haemopurulent fluid were encountered with evidence of communication with the hip joint. Three days post-operatively, pus obtained at the time of initial aspiration showed profuse growth of grey–white colonies only on the anaerobic plates at day 3 of incubation. The organism was identified as *C. difficile* by Bruker MALDI-TOF MS with a score of 2.34, confirming confident identification of the organism to the species level (score >2.0 utilizing standard Bruker interpretative criteria). The patient was subsequently switched to IV vancomycin (weight-based dosing) and metronidazole 500 mg TDS. In the following weeks, due to rising CRP, ongoing fevers and re-accumulation of fluid, four open surgical debridement procedures were performed with the aim to achieve source control and to perform intraoperative sampling. *C. difficile* was identified in multiple tissue samples and aspirates of joint fluid, confirming the diagnosis of a prosthetic joint infection due to *C. difficile*; blood cultures collected remained negative throughout this time. Antimicrobial susceptibility testing was performed and interpreted at the reference laboratory using the European Committee on Antimicrobial Susceptibility Testing (EUCAST) version 13.0 (2023) published MIC breakpoints. The isolate was susceptible to vancomycin (MIC, 0.38 mg l^−1^) and metronidazole (MIC, 0.125 mg l^−1^); additional antibiotics including doxycycline, penicillin and clindamycin were also tested, for which no breakpoints were available, limiting interpretation (see Table 1).

**Table 1.** Susceptibility profile

Antibiotic	EUCAST MIC (mg/L)	Interpretation
Clindamycin	4 mg l^−1^	No interpretation available
Metronidazole	0.125 mg l^−1^	Sensitive
Penicillin	2 mg l^−1^	No interpretation available
Tetracycline	0.064	No interpretation available
Vancomycin	0.38	Sensitive
Ciprofloxacin	>32	No interpretation available

Given the challenge in controlling the infection, there was concern about a potential fistula between the hip joint and colon, leading to ongoing contamination of the joint. However, an unremarkable CT scan of the abdomen and pelvis and multiple stool samples that tested negative for *C. difficile* toxin excluded this. Additionally, the possibility of a polymicrobial infection was considered as a contributing factor to the lack of improvement. To investigate further, we sent joint fluid for metagenomic analysis, and whole-genome sequencing (WGS) was performed on two *C. difficile* isolates, obtained 3 weeks apart, to assess their virulence factors and resistance profiles. Metagenomic analysis of the sequencing data from the DNA extract of the hip fluid by k-mer-based approaches (using the Kraken2 and OneCodex bioinformatic tools) detected a low number of *C. difficile* sequences, but no other pathogen sequences were detected at significant levels.

Due to the patient’s ununited fracture and the extent of the initial fracture fixation, curative surgical intervention was deemed not achievable – therefore, thorough and repeated surgical debridement was pursued, with the ultimate aim of achieving long-term antibiotic suppression of infection. The patient received a total of 6 weeks of metronidazole (2 weeks IV followed by 4 weeks PO) before being switched to PO doxycycline 100 mg twice daily (BD) as suppressive therapy. At the time of writing, the patient had shown a sustained response to therapy for >12 months and continued to be reviewed by the Infectious Diseases team.

## Sequencing methods

WGS and bioinformatics analysis using k-mer-based species identification. DNA was extracted from the isolates using the DSP DNA Mini Kit (Qiagen, Germantown, Maryland, USA) on the QIASymphony, and DNA from the fluid sample was extracted using the MasterPure Complete DNA and RNA purification kit (BioSearch Technologies, LGC Group, Teddington, Middlesex, UK). The DNA libraries for the samples and for an extraction control were prepared as per the Illumina Nextera XT protocol (Illumina, San diego, California, USA) and sequenced on the NextSeq500 according to the manufacturer’s protocol.

Human sequences were removed from the sequence reads, and the remaining sequences were analysed using two k-mer-based methods: Kraken (v2.0.6) against the Minikraken2 database (updated on 26 November 2021) and OneCodex using the OneCodex database (accessed on 08 September 2023).

## Literature review

A literature review was performed by searching the PubMed database, followed by trawling the reference lists of identified articles. Seven previous cases of prosthetic or fixation-related bone or joint infections due to *C. difficile* were identified (see Table 2) [2,8]. The most common joint involved was the hip (4/7), followed by knee (2/7) and shoulder (1/7). Five patients underwent surgical intervention involving implant removal. Despite this, two cases went on to require amputation. Only one case was successfully managed with debridement and implant retention (DAIR). In this instance, the patient presented with an early post-operative infection and the patient underwent surgical intervention within 7 days of their index arthroplasty [7].

**Table 2.** Literature review

	Case 1	Case 2	Case 3	Case 4	Case 5	Case 6	Case 7
**References**	[2]	[3]	[4]	[5]	[6]	[7]	[8]
**Age and sex**	83F	16M	31F	75F	47F	61M	61F
**Onset (time**)	12 months	16 months	>12 months	5 years post-revision	3 months	1 week	5 weeks post-second-stage revision
**Site of prosthesis**	Hip (pin and plate for femoral fracture)	Knee arthroplasty	Left hip arthroplasty	Right hip arthroplasty	Left shoulder arthroplasty	Left hip arthroplasty	Left knee arthroplasty
**Origin of infection**	Post antibiotic-associated diarrhoea	Post patella fracture and sterile joint aspiration	Post antibiotic-associated diarrhoea	Unknown	Unknown	Haematogenous spread via intestinal translocation	Inoculation at the time of surgery or post-operatively or haematogenous spread from intestinal tract
**Comorbidities**	Unknown	Osteosarcoma of knee, recent chemotherapy	Sickle cell disease, bilateral avascular necrosis femoral heads	Unknown	Hypothyroidism Alcoholic hepatitis	HIV, CMV disease, diabetes mellitus	Nil
**Bacteraemia**	No	No	No	No	No	Yes	No
**Diarrhoea**	Yes	No	Yes	No	No	No	No
**Toxin status**	Unknown	Nontoxigenic	Unknown	Toxigenic (PCR pos *tcdA* and *tcdB* genes)	Unknown	Nontoxigenic	Toxigenic (PCR pos *tcdC* gene)
**Surgical intervention**	Removal of hardware and revision	Removal of implant after failing arthrotomy with drainage	Incision and drainage of periprosthetic abscess	Two-stage revision with the use of vancomycin cement spacer	Surgical debridement with single-stage revision and antibiotic spacer	DAIR	Debridement and removal of implant
**Antibiotics used**	Metronidazole	Amoxicillin/ ornidazole/ rifampicin/ lincomycin/ penicillin G	Metronidazole	Metronidazole	Vancomycin/ metronidazole	Vancomycin/ metronidazole	Piperacillin-tazobactam/ metronidazole
**Outcome**	Unknown	Amputation	Death	No relapse at 12-month follow-up	Unknown	No relapse at 2-year follow-up	Amputation

Interestingly, only two of the seven previous cases occurred following a definite diarrhoeal illness, with five of seven cases occurring without evidence to suggest a preceding episode of *C. difficile* colitis. All cases were managed with antibiotic regimens containing metronidazole. Of the four cases that underwent *C. difficile* toxin testing, two isolates were found to be non-toxigenic, with *tcdA* and *tcdB* genes detected by PCR in one isolate and the *tcdC* gene detected by PCR also in one isolate. No isolates previously described in the literature have undergone WGS.

## Discussion

*C. difficile* is an anaerobic, ubiquitous, spore-forming rod. It is widely present in the environment, with reservoirs including asymptomatic and symptomatic carriers, animal intestinal tracts (canine, feline, porcine) and contaminated surfaces [9]. Intestinal infection, including diarrhoea and pseudomembranous colitis, is the most common clinical manifestation, and as such, the ability of *C. difficile* to cause colonic disease through enzyme and toxin production is well described [10]. Extra-colonic manifestations are extremely rare, though those most commonly reported include small intestine infiltration, reactive arthritis and bacteraemia [9,10]. Osteoarticular infections, including prosthetic joint infections, account for a minority of extra-colonic manifestations, and the pathogenesis by which *C. difficile* can do so is poorly understood. Given the timing of onset and chronicity of the patient’s symptoms (>4 weeks), the exclusion of an enteric fistula via CT imaging, the repeated faecal contamination in close proximity to the surgical site prompting frequent dressing changes and the prolonged survival of *C. difficile* spores on the patient’s skin and in the environment, the authors propose that direct wound inoculation was the most likely route of acquisition of *C. difficile* into the joint. Haematogenous spread from a transient episode of bacteraemia in the setting of colitis remains an alternative explanation and has been implicated as a route of transmission in previous cases of extra-colonic *C. difficile* infection (CDI) [11].

The underlying pathogenesis of *C. difficile*-related extraintestinal infections remains unknown. Nevertheless, the pathogenic behaviour of this organism can be extrapolated from its pathology in *C. difficile* colitis. The major virulence factors produced by *C. difficile* are the exotoxins tcdA and tcdB, which are both enterotoxic and cytotoxic. Deletions in the gene *tcdC*, a negative regulator of toxins A and B, lead to overproduction of these virulence factors. Toxins A and B induce cytoskeletal alterations, leading to the breakdown of the tight junctions of the epithelial connection. Translocation of these cytotoxic toxins into the cells triggers inflammation and ultimately results in diarrhoea and pseudomembranous colitis [12]. *C. difficile* transferase or binary toxin is a third toxin produced by some *C. difficile* strains. While its exact role remains undefined, several clinical studies suggest an association between its prevalence in strains commonly associated with severe CDI, including the epidemic B1/NAP1/027 and ribotype (RT) 078 isolates [13]. While toxins are the primary virulence factors of *C. difficile*, it is crucial to acknowledge the significant role played by other putative virulence factors in adherence and colonization. These factors, including the surface layer and cell wall proteins (CWPs), fibronectin-binding proteins, flagella, fimbriae and the heat shock protein GroEL, are often overshadowed by the potency of the toxins in disease, yet numerous studies have highlighted their role in the pathogenesis of *C. difficile* [14].

While managing this case, we had several questions about the potential pathogenesis of *C. difficile* in extraintestinal disease, primarily whether these strains acquired additional virulence factors to thrive outside their usual enteric niche. Therefore, we sequenced two of our patient’s isolates to understand the epidemiology and virulome profile (Tables3  4). Our isolates had toxin B and binary toxin genes detected but did not harbour the *tcdC* mutation associated with hypervirulence. Upon comparison with previously sequenced faecal isolates locally, no novel or unusual virulence factors were detected in our isolates. Interestingly, we found that virulence factor CD3246 was found in only one isolate (M230583, isolated on 1 July 2023), but not in the other (M2305774, isolated on 23 July 2023). Studies have shown the involvement of CD3246 in early biofilm formation in * C. difficile* along with other cell surface proteins CD0183, CD2831 and CD3392 [15]. A plausible reason for not detecting CD3246 in the second isolate collected late in the course of illness is potentially due to the loss of gene for one of the biofilm-forming virulence factors, which may have resulted from exposure to vancomycin and metronidazole. Penesyan *et al*. [16] investigated the microevolution of biofilm cells in *Acinetobacter baumannii* in response to antibiotic exposure and found that these cells showed a higher incidence of insertion sequence-mediated mutations and, in some cases, plasmid loss compared to antibiotic-free controls. Metronidazole is also hypothesized to stimulate mutations and recombination in *Helicobacter pylori*, thereby impacting host-specific adaptation and the evolution of virulence [17]. Another study on biofilm regulation in *C. difficile* highlighted significant variations in flagellar gene expression with antibiotics and other stressors, supporting our hypothesis [18].

**Table 3.** MLST and virulence analysis

Sample identifier and date of collection	MLST	Toxin A (toxB)	Toxin B	TcdC	Binary toxin	CD0873	CD2831	CD3246	cdtA	cdtB	cwp66	cwp84	fbpA/fbp68	groEL	zmp1
M2305835 (23 July 2023)	ST55	nd	d	wt	d	d	d	nd	d	d	d	d	d	d	d
M2305774 (01 July 2023)	ST55	nd	d	wt	d	d	d	d	d	d	d	d	d	d	d

d, detected; MLST, multilocus sequence typing; nd, not detected; wt, wild type.

**Table 4.** Virulence genes and their proposed functions

Gene	Product
CD0873	(CD0873) ABC transporter substrate-binding protein [CD0873 (VF0593) – Adherence (VFC0001)] [*C. difficile* 630]
CD2831	(CD2831) SrtB-anchored collagen-binding adhesin [CD2831 (VF0598) – Adherence (VFC0001)] [*C. difficile* 630]
CD3246	(CD3246) Cys-Gln thioester bond-forming surface protein [CD3246 (VF0599) – Adherence (VFC0001)] [*C. difficile* 630]
cdtA	(cdtA) CdtA [CDT (VF0385) – Exotoxin (VFC0235)] [*C. difficile* 630]
cdtB	(cdtB) CdtB [CDT (VF0385) – Exotoxin (VFC0235)] [*C. difficile* 630]
cwp66	(cwp66) cell wall-binding protein Cwp66 [Cwp66 (VF0591) – Adherence (VFC0001)] [*C. difficile* 630]
cwp84	(cwp84) cell wall-binding cysteine protease Cwp84 [Cwp84 (VF0590) – Exoenzyme (VFC0251)] [*C. difficile* 630]
fbpA/fbp68	(fbpA/fbp68) fibronectin-binding protein FbpA [FbpA/Fbp68 (VF0595) – Adherence (VFC0001)] [*C. difficile* 630]
groEL	(groEL) chaperonin GroEL [GroEL (VF0594) – Adherence (VFC0001)] [*C. difficile* 630]
toxB	(toxB) toxin B [TcdB (VF0377) – Exotoxin (VFC0235)] [*C. difficile* 630]
zmp1	(zmp1) zinc metalloprotease Zmp1 [Zmp1 (VF0600) – Exoenzyme (VFC0251)] [*C. difficile* 630]

Furthermore, our isolates did not exhibit antimicrobial resistance (AMR) genes, aligning with the phenotypic susceptibility profile. This finding is consistent with the previous Australian surveillance data and the sequencing results of recurrent CDI cases from Western Australia [19]. Historically, Australia has had a low prevalence of resistance due to the conservative use of fluoroquinolones [20]. AMR, in general, due to the use of broad-spectrum antibiotics, especially fluoroquinolones, third-generation cephalosporins and clindamycin, is a crucial driver of *C. difficile* epidemiology. CDI outbreaks are linked to the evolution of resistance to clindamycin (RT017), fluoroquinolones (RT027) and tetracycline (RT078). Studies have shown a strong association between the major epidemic strains of *C. difficile* and specific AMR determinants, giving them a survival edge against antibiotics while incurring minimal fitness costs [21].

In addition to the rarity of the implicated organism, our patient presented multiple challenges in terms of surgical management. The chronic nature of their symptoms, combined with microbiological factors and patient comorbidities, suggested that an exchange arthroplasty would be the intervention most likely to achieve a cure of infection. However, due to the presence of an ununited femoral fracture, the extent of femoral fixation and significant bone loss, it was determined that aggressive debridement with implant and fixation retention was the only feasible surgical option, aside from amputation. Whilst in selected patients, long-term antibiotic suppression of a prosthetic joint infection to maintain a functioning prosthesis can be successful [22,23], our case was complicated by a lack of viable long-term antibiotic options to achieve suppression. Prolonged administration of metronidazole raised concerns for increased risk of neurotoxicity including irreversible peripheral neuropathy [24], and PO vancomycin or fidaxomicin has insufficient systemic absorption to be effective outside of the gut [25]. Thus, we chose doxycycline based on the isolate’s *in vitro* susceptibility testing (MIC, 0.064 mg l^−1^), pharmacokinetic profile and reduced likelihood of causing further episodes of *C. difficile* colitis compared to other antimicrobials [26,27].

## Conclusion

This case highlights the rare and complex nature of prosthetic joint infections caused by *C. difficile*. Our experience underscores the critical role of a multidisciplinary approach in managing these infections, including optimizing surgical interventions, selecting antibiotics based on both susceptibility profiles and patient tolerability and employing advanced diagnostic tools like WGS. The sequencing of this patient’s isolates provided valuable insights into the pathogen’s virulence factors and resistance profile, though further research is essential to deepen our understanding of *C. difficile’s* extracolonic pathogenesis.

The successful suppression of this patient’s infection with doxycycline – an antibiotic not typically used for colonic CDIs – demonstrates its potential for long-term management when curative surgical options are limited. This case adds to the scarce literature on extraintestinal CDIs, underscoring the need for ongoing research and case reporting to enhance our understanding of the pathogenesis and optimal treatment of these rare occurrences.

## Abbreviations

AMR: antimicrobial resistance
CDI: *Clostridioides difficile* infection
CRP: C-reactive protein
CT: computed tomography
CWP: cell wall protein
DAIR: debridement and implant retention
EUCAST: European Committee on Antimicrobial Susceptibility Testing
IV: intravenous
PJI: prosthetic joint infection
PO: oral
RT: ribotype
TDS: three times a day
THR: total hip replacement
WGS: whole-genome sequencing
